# The Relationships Among Transverse Sinus Stenosis Measured by CT Venography, Venous *Trans*-stenotic Pressure Gradient and Intracranial Pressure in Patients With Unilateral Venous Pulsatile Tinnitus

**DOI:** 10.3389/fnins.2021.694731

**Published:** 2021-09-03

**Authors:** Xiaoyu Qiu, Pengfei Zhao, Xiaoshuai Li, Heyu Ding, Han Lv, Rong Zeng, Guopeng Wang, Long Jin, Zhenghan Yang, Shusheng Gong, Zhenchang Wang

**Affiliations:** ^1^Department of Radiology, Beijing Friendship Hospital, Capital Medical University, Beijing, China; ^2^Department of Otolaryngology Head and Neck Surgery, Beijing Friendship Hospital, Capital Medical University, Beijing, China; ^3^Department of Interventional Radiology, Beijing Friendship Hospital, Capital Medical University, Beijing, China

**Keywords:** pulsatile tinnitus, intracranial hypertension, transverse sinus stenosis, pressure, CT venography

## Abstract

**Objectives:**

To assess a non-invasive means of predicting a venous *trans*-stenotic pressure gradient (TPG) and intracranial pressure (ICP) as opposed to invasive examinations in unilateral venous pulsatile tinnitus (PT) patients.

**Methods:**

Thirty patients with unilateral venous PT who presented symptomatic-sided transverse sinus stenosis (TSS) on computed tomography venography (CTV), ipsilateral TPG measured by digital subtraction angiography (DSA) and cerebrospinal fluid (CSF) pressure measured by lumbar puncture were included. The ratio of TSS was calculated by dividing the cross-sectional areas of the maximal stenosed transverse sinus by that of the adjacent normal transverse sinus on CTV. The correlations among and predictive values of TSS, TPG, and ICP were analyzed.

**Results:**

In patients with unilateral venous PT, the symptomatic-sided and average bilateral TSS values were 78 ± 11 and 77 ± 9%; ICP, 230.50 ± 55.75 mmH_2_O; and the TPG, 9.51 ± 5.76 mmHg. The symptomatic-sided TSS was linearly and positively correlated with TPG (*R*^2^ = 0.400), and the symptomatic-sided and bilateral average TSS both showed weak correlations with ICP (*R*^2^ = 0.288, *R*′^2^ = 0.156). When the degree of TSS increased by 10%, the TPG and ICP increased by approximately 3.3 mmHg and 25.8 mmH_2_O, respectively. The receiver operating characteristic curve showed the optimal threshold of ipsilateral TSS for a positive TPG was 0.75, while TSS had no significant predictive value for ICP (*p* > 0.05). TPG and ICP also exhibited a linear positive correlation (*R*^2^ = 0.552). When ICP increased by 10 mmH_2_O, the TPG increased by approximately 0.77 mmHg, and the optimal threshold of ICP for a positive TPG was 227.5.

**Conclusion:**

TSS, TPG, and ICP are interrelated. TSS measured by CTV can predict TPG in patients with unilateral venous PT.

## Introduction

Pulsatile tinnitus (PT) typically manifests as a pulse-synchronous sound in the ear, the long-term presence of which seriously reduces sufferers’ life and work quality. Most cases are found to have a venous origin after comprehensive examinations (Schoeff et al., 2014). Venous PT and idiopathic intracranial hypertension (IIH) are closely related. On the one hand, IIH is a common cause of venous PT; on the other hand, PT is a common or even initial symptom of IIH (Markey et al., 2016; Guo et al., 2019).

Transverse sinus stenosis (TSS) is a common feature of both venous PT and IIH (Markey et al., 2016; Hewes et al., 2020). The *trans*-stenotic pressure gradient (TPG) formed by TSS may be a common pathological basis for these conditions. Current studies suggest that PT may originate from strong and jet-like blood flow impacts caused by transverse sinus TPG (Amans et al., 2018; Li et al., 2018) and that it may also cause obstruction of cerebrospinal fluid (CSF) drainage through arachnoid granules into sinuses, leading to IIH (Funnell et al., 2018; Dinkin and Oliveira, 2019).

Recognizing the presence of elevated intracranial pressure (ICP) and positive TPG is important when evaluating patients with unilateral PT and developing a treatment strategy. Patients with sigmoid sinus wall anomalies may benefit from surgical reconstruction (Eisenman et al., 2018; Hsieh and Wang, 2020), whereas patients with TSS and TPG can be treated with endovascular stenting (Cuellar et al., 2018; Lenck et al., 2019). Notably, TSS may be an asymptomatic finding in PT patients without IIH, in whom stenting should be avoided. For patients with IIH, weight loss and drugs reducing ICP are first preferred, but for IIH patients who do not respond to medical therapy, TPG ≥ 8 mmHg is typically considered an indication for stenting (Levitt et al., 2017; West et al., 2019). However, most PT patients have no typical clinical symptoms of IIH (Hewes et al., 2020). Although IIH has been widely reported to be associated with bilateral TSS, a clear correlation between the degree of TSS and features of ICP has not been established (Riggeal et al., 2013). Recently, Hewes et al. (2020) used the method of TSS diameter measurement and combined conduit score to evaluate TSS and suggested that the degree of TSS in some PT patients was greater than that noted in normal individuals but lower than that of IIH patients. However, due to the irregular shape of the TSS, the diameter may not accurately reflect the degree of TSS, which affects the accuracy of the final results.

*Trans*-stenotic pressure gradient measurement relies on endovascular venous manometry. Digital subtraction angiography (DSA) is currently used for diagnosing mild vascular abnormalities in PT patients (Lenck et al., 2019). Given its invasiveness, DSA is not routinely performed, and manometry is not attempted except for the proposed stenting. Although the pathophysiology of TSS, ICP, and TPG are closely related, there is still a lack of relevant research. In Levitt et al. (2017) first reported that a positive TPG was found in 35.4% of IIH patients but 0% of patients without IIH. West et al. (2019) suggested that TPG was more correlated with the degree of TSS measured by DSA than with that measured by MR venography/computed tomography venography (CTV) with TPG increasing by 3.5 mmHg for every 10% increase in TSS.

Computed tomography venography is recommended for the evaluation of PT patients, and this method can be used to evaluate most PT etiologies in a single examination (Krishnan et al., 2006; Zhao et al., 2016). Based on the close relationships among TSS, TPG, and ICP, we hypothesized that quantitative correlations exist among them. In this study, we analyzed the data of 30 patients with unilateral venous PT who underwent CTV, venous manometry and lumbar puncture to explore their possible relationships.

## Materials and Methods

### Patients

**FIGURE 1:**
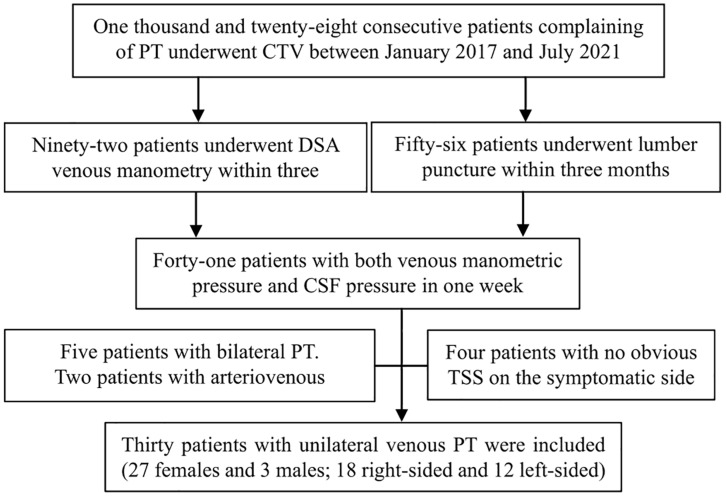
The inclusion process applied in this study. PT, pulsatile tinnitus; CTV, computed tomography venography; DSA, digital subtraction angiography; CSF, cerebrospinal fluid; TSS, transverse sinus stenosis.

After obtaining Ethics Committee approval, thirty patients who complained of unilateral PT from January 2017 to July 2021 were retrospectively included. Unilateral PT was defined as the patient’s complaint that there was beating tinnitus in the unilateral ear that was consistent with the frequency of the heartbeat/pulse, and compression of the affected side of the neck could eliminate or obviously alleviate the sound. The criteria for inclusion in the database were as follows: (1) CTV examination and (2) Endovascular venous manometry and lumbar puncture were performed for CSF measurement within 3 months. The exclusion criteria were as follows: (1) bilateral PT; (2) PT inconsistent with the heartbeat; (3) no improvement after compression of the jugular vein on the symptomatic side; (4) TSS < 50% on CTV; and (5) venous sinus thrombosis, neoplasms, or arterial/arteriovenous abnormalities. Written informed consent was signed before every examination for each patient. The inclusion process is shown in Figure 1.

### Assessment Methods

#### Computed Tomography Venography Examination and Evaluation

**FIGURE 2:**
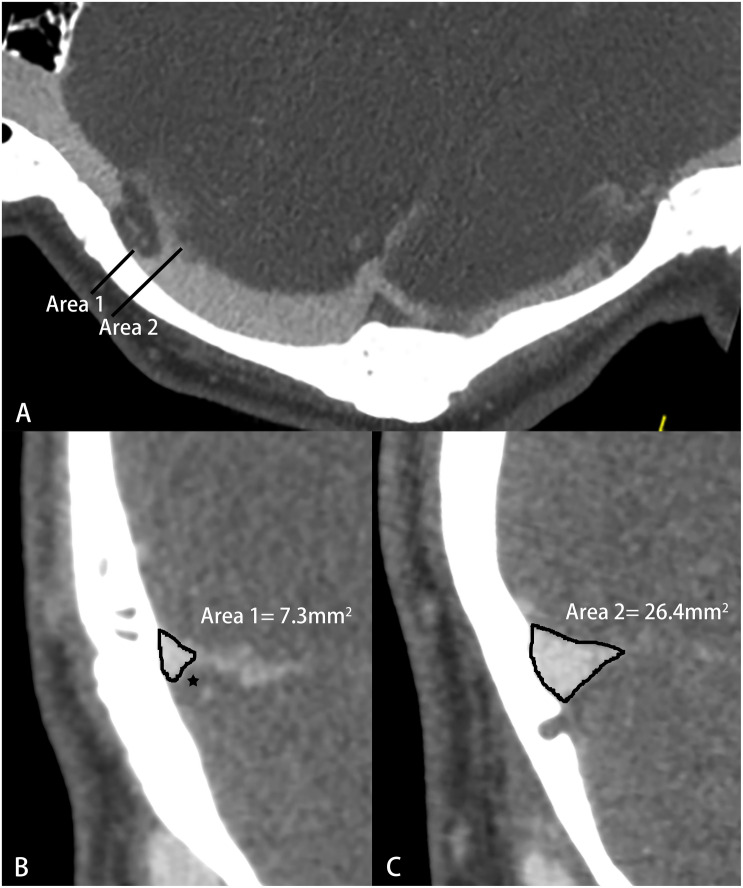
Schematic diagram of transverse sinus stenosis (TSS) measured by computed tomography venography (CTV). **(A)** Curve planar reformation image; **(B)** The most severe cross section of TSS, ★-arachnoid granulation; **(C)** Normal transverse sinus cross-sectional area.

Computed tomography venography images were obtained by a 64- or 256-spiral CT scanner (Brilliance, Philips Healthcare; Revolution, GE Healthcare). The imaging parameters were as follows: 120 kV, automatic milliampere s; matrix 512 × 512, collimation width 64 or 256 × 0.625 mm, rotation time 0.5 s, pitch 0.992:1; contrast (iopamidol, Bracco Diagnostics), 370 mg iodine/ml; 1.5 ml/kg, 5 ml/s. All images were delivered to a postprocessing workstation (Extended Brilliance Workspace, Philips Healthcare). Curve planar reformation was used to show the whole course of the bilateral transverse sinuses. The cross-sectional area of the largest bilateral TSS for each patient was measured (Figure 2). Any transverse sinus with multiple stenoses was measured at the most severe cross-section. The normal areas of the left and right transverse sinuses were measured at the cross-section adjacent to the largest TSS. The percentage of symptomatic-side TSS and average TSS were calculated using the formulas: 1 – (symptomatic-side TSS area/ipsilateral normal area) and 1 – (left TSS area + right TSS area)/(left normal area + right normal area), respectively. The average TSS was calculated to provide a single measure of the average percent TSS for all patients (Zhao et al., 2016).

#### Endovascular Venous Manometry

Digital subtraction angiography was performed with Seldinger’s technique using an angiography machine (Innova 4100-iq, GE Healthcare) after local anesthesia. First, percutaneous femoral artery puncture was performed to observe the images of venous sinuses, especially ipsilesional TSS. Then, through femoral vein puncture, a 5F catheter and 2.7F coaxial microcatheter (Stride, Asahi) were introduced to the internal jugular vein and intracranial venous sinus, respectively, and pressure sensors (DPT- 248, Yixinda) were connected. The tip of the microcatheter was placed at the distal side of the ipsilateral transverse sinus and the proximal side of the sigmoid sinus. TPG was calculated as the difference in mmHg, and more than 8 mmHg was positive.

#### Cerebrospinal Fluid Pressure Measurement

Lumbar puncture was performed in the lateral decubitus manometry position after local anesthesia was induced. The stabilized opening pressure was recorded after the patient was totally relaxed. ICP ≥ 250 mmH_2_O indicated elevated ICP (Friedman et al., 2013; Markey et al., 2016). All patients with IIH underwent lumbar puncture, determination of body mass index (BMI), and neurological and ophthalmic examinations, and met the diagnostic criteria of IIH (Friedman et al., 2013).

### Statistical Analysis

SPSS 24 (IBM, United States) was used for descriptive statistics, linear regression analysis, and ROC analysis, and Medcalc 15 (MedCalc Software Ltd., Belgium) was used to calculate the predicted value and 95% confidence interval. The mean ± standard deviation was presented for normally distributed variables, and the independent sample *t*-test was used to compare the differences between each pair of groups. The correlations among TSS, TPG, and ICP were evaluated by univariate linear regression analysis. For relationships with statistical significance, the optimal threshold and area under the curve were analyzed based on the receiver operating characteristic (ROC) curve, and the sensitivity, specificity, predictive value, and 95% confidence interval of the values for predicting elevated ICP and positive TPG were calculated based on the optimal thresholds. Here, *p* < 0.05 was considered statistically significant.

## Results

### Descriptive Statistics

A total of 30 PT patients (3 males and 27 females, 18 right-sided and 12 left-sided) were included. The average age was 36.67 ± 9.54 years. The mean BMI was 30.44 ± 1.16. The percentages of symptomatic-sided and average TSS were approximately 78 ± 11 and 77 ± 9%, respectively. The mean ICP was 230.50 ± 55.76 mmH_2_O. In total, 14 patients (46.7%) with elevated ICP were clinically confirmed as IIH, and 16 (53.3%) did not have elevated ICP. The mean value of TPG was 9.51 ± 5.76 mmHg. Eighteen patients (60%) were TPG-positive, and 12 (40%) were TPG-negative.

### The Relationship Between TSS and TPG

The symptomatic-sided TSS was linearly positively correlated with TPG (*R*^2^ = 0.400, *p* = 0.000), but there was no correlation between bilateral average TSS and TPG (*R*′^2^ = 0.072, *p*′ = 0.153). The univariate linear regression analysis for TSS on the symptomatic side and TPG was as follows: *y*_TPG_ = −15.89 + 32.73x_TSS_ (*p* = 0.000), suggesting that the degree of TSS increased by 10% and that TPG increased by approximately 3.3 mmHg. The ROC curve showed that the optimal threshold of ipsilateral TSS for the prediction of TPG was 0.75 (AUC = 0.870, Youden index = 0.778, *p* = 0.001), and the sensitivity and specificity were 94 and 83%, respectively, as shown in Table 1.

**TABLE 1:** Diagnostic accuracy of intracranial pressure (ICP) elevation and *trans*-stenotic pressure gradient (TPG) positivity in 30 patients with unilateral venous pulsatile tinnitus (PT).

		TPG+	TPG−	Sens		Spec		PPV		NPV	
				%	95% CI	%	95% CI	%	95% CI	%	95% CI
TSS	≥75%	17	2	94	72.7–99.9	83	51.6–97.9	90	66.9–98.7	91	58.7–99.8
	<75%	1	10								
ICP	>227	15	1	92	61.5–99.8	83	58.6–96.4	94	69.8–99.8	79	49.2–95.3
	≤227	3	11								

*TSS, transverse sinus stenosis; IIH, idiopathic intracranial hypertension; PPV, positive predictive value; NPV, negative predictive value.*

### The Relationship Between TSS and Elevated ICP

Symptomatic-sided TSS and average TSS were both positively correlated with ICP, but the correlation was weak. The univariate linear regression analysis was as follows: *y*_ICP_ = 21.85 + 268.88x_TSS__1_ (*R*^2^ = 0.288, *p* = 0.002); *y*′_ICP_ = 31.69 + 257.75x_TSS__2_ (*R*′^2^ = 0.156, *p*′ = 0.031). However, the ROC curve showed that the two parameters had no significant predictive value for elevated ICP (AUC = 0.665, Youden index = 0.420, *p* = 0.124; AUC′ = 0.583, Youden index′ = 0.205, *p*′ = 0.442).

### The Relationship Between ICP and TPG

Intracranial pressure was linearly positively correlated with TPG. The linear regression analysis was as follows: *y*_TPG_ = −8.18 + 0.077x_ICP_ (*R*^2^ = 0.552, *p* = 0.000). The optimal threshold of ICP for the prediction of TPG was 227.5 (AUC = 0.924, Youden index = 0.750, *p* = 0.000), and the sensitivity and specificity were 92 and 83%, respectively, as shown in Table 1.

## Discussion

Computed tomography venography can serve as a non-invasive tool to predict TPG, which may reduce the need for endovascular manometry in selected patients. Due to the irregular shape of the TSS, CT curved planar reformation was used to measure the maximum cross-sectional area and calculate the percentage to quantitatively reflect the degree of TSS. This process is different from those used in most previous association studies. The percentage of the TSS width measured by Riggeal et al. (2013) and diameter grading of TSS with combined conduit score described by Hewes et al. (2020) and Farb et al. (2003) have been used. These methods are mainly based on the TSS diameter measurements. The average percent TSS for each patient was also counted to reflect the degree of dural venous outflow obstruction with reference to the method of Riggeal et al. (2013). The results showed that symptomatic-sided TSS was correlated with TPG compared with average TSS. Compared with the average TSS, the symptomatic-side TSS has a stronger correlation with ICP; however, neither symptomatic-sided TSS nor average TSS can be used to predict ICP, which is consistent with previous research conclusions (Riggeal et al., 2013; Dinkin and Oliveira, 2019). These data suggest that other factors may influence ICP in addition to TSS, including TPG, venous collateral circulation or lymphatic drainage. Moreover, lumbar puncture is the gold standard for the clinical measurement of ICP, so it plays an irreplaceable role.

The presence of TPG and jet-like rapid blood flow are important for determining whether TSS is symptomatic. Our results show that TPG increased by 3.3 mmHg for every 10% increase in TSS, which is similar to the results reported by West et al. (2019). They reported that when the degree of stenosis increased by approximately 10%, TPG increased by approximately 3.5 mmHg (West et al., 2019), suggesting that the degree of TSS can be used as a predictor for TPG. However, unlike their conclusion, our results showed that the best predictive value of TSS for TPG was 75%, which was greater than the 34% reported by previous authors who used DSA. This finding may be related to the use of different methods. First, patients with TSS < 50% were not included in this study. Second, the area of TSS was measured by CTV rather than the diameter measured by DSA.

Moreover, the results of our study suggest that TSS based on CTV, which is similar to that based on DSA, exhibits high accuracy in predicting TPG positivity, suggesting that CTV may be used to select which patients should undergo catheter manometry and identify patients who are unlikely to have TPG and therefore do not need this invasive examination.

In addition, for PT patients with identified IIHs who are clearly planning for stenting, our results suggest that both ICP and TSS can be used to predict the possibility of TPG positivity; however, the correlation is stronger for ICP than for TSS. For every 10 mmH_2_O increase in ICP, TPG increased by 0.77 mmHg. Although lumbar puncture is an invasive examination, it is the “gold standard” for the clinical evaluation of ICP. Predicting TPG levels by ICP measured by lumbar puncture can reduce the frequency of invasive DSA examinations.

This study has several limitations. First, the sample size was slightly small. Although the number of PT patients is increasing, only a few patients underwent simultaneous DSA and lumbar puncture because of psychological resistance to invasive operations. However, our preliminary results show that TPG and IIH exhibit fairly high predictive values. These factors can be used as an aid for screening, reduce or even avoid further invasive examinations, and help to develop treatment strategies. Second, all patients in this study were clinically suspected having IIH and subsequently underwent lumbar puncture pressure manometry. Thus, the characteristics of PT patients without IIH in this study may represent only those with an elevated risk of ICP and may not necessarily be generalizable to the general population. In addition, the reliability in patients without PT should be further verified.

## Conclusion

The results of this study show that in patients with unilateral venous PT, symptomatic-sided TSS, ICP, and TPG exhibit linear positive correlations. When determined based on CTV, the degree of TSS can be used to predict TPG. Therefore, CTV can be used to screen whether other invasive examinations are needed in the next step. Moreover, ICP can predict TPG, and the result of a “gold standard” invasive method can be used to predict whether another invasive examination is needed.

## Data Availability Statement

The original contributions presented in the study are included in the article/supplementary material, further inquiries can be directed to the corresponding authors.

## Ethics Statement

The studies involving human participants were reviewed and approved by the Beijing Friendship Hospital, Capital Medical University. The patients/participants provided their written informed consent to participate in this study.

## Author Contributions

XQ, PZ, XL, HD, and HL performed the experiment and collected, analyzed, or interpreted the data involved in the study. XQ and PZ preprocessed the imaging data, performed the statistical results, and drafted the manuscript. RZ, GW, LJ, and SG collected the clinical data. ZW and SG designed the study and ensured the questions related to all aspects of the work. PZ, ZY, SG, and ZW provided the critical comments on the manuscript. All authors contributed to the article and approved the submitted version.

## Conflict of Interest

The authors declare that the research was conducted in the absence of any commercial or financial relationships that could be construed as a potential conflict of interest.

## Publisher’s Note

All claims expressed in this article are solely those of the authors and do not necessarily represent those of their affiliated organizations, or those of the publisher, the editors and the reviewers. Any product that may be evaluated in this article, or claim that may be made by its manufacturer, is not guaranteed or endorsed by the publisher.
